# Pneumatosis Intestinalis of the Colon and Greater Omentum following Small Bowel Resection

**DOI:** 10.1155/2022/2670244

**Published:** 2022-04-16

**Authors:** Matthew G. K. Benesch, Mark F. O'Driscoll

**Affiliations:** Discipline of Surgery, Faculty of Medicine, Memorial University of Newfoundland, St. John's, NL, Canada A1B 3V6

## Abstract

**Introduction:**

Pneumatosis intestinalis (PI) is a condition of gas collection within the bowel wall that can represent either a benign clinical finding or a forerunner to potential gastrointestinal catastrophe. As a potentially sinister discovery typically first detected on radiographic imaging, clinicians need to astutely assess the need for additional urgent medical or surgical management in these patients. Apart from portal venous gas, PI outside of the bowel wall is an extremely rare entity that is poorly described. Hence, it is not necessarily clear if PI outside the bowel wall warrants more aggressive management. *Case Presentation*. We describe a patient with intermittent abdominal pain who presented with PI of the greater omentum in addition to the right and transverse colon nearly two weeks after small bowel resection. Due to his clinical stability, we elected to closely observe him. His condition completely resolved with conservative management. *Discussion*. PI in the omentum has not been described in a patient who has survived their underlying pathology. Our patient demonstrated PI radiographically in his right and transverse colon and omentum with complete resolution. We did not have to alter our clinical management because of this unique clinical presentation.

**Conclusion:**

This case highlights that pneumatosis intestinalis can extend extraluminally and still be managed conservatively with judicious monitoring in the otherwise stable patient.

## 1. Introduction

Pneumatosis intestinalis (PI), also known as pneumatosis cystoides intestinalis, pneumatosis coli, intramural gas, pseudolipomatosis, intestinal emphysema, or lymphopneumatosis, is a multifactorial and poorly understood phenomenon where gas accumulates within the wall of the small or large intestine [1]. PI is sometimes an incidental finding but in some clinical circumstances it foreshadows a life-threatening intra-abdominal condition, particularly in the context of peritonitis, metabolic acidosis, and presence of portal venous gas [2]. The pathogenesis of PI has been proposed to be through either mechanical, bacterial, or biochemical processes, all of which contain features that force gas through mucosal breaks or disruptions into the submucosa [3, 4]. Approximately 42 percent of cases of PI involve the small bowel, 36 percent the colon, and 22 percent involve both the small bowel and colon [5]. Clinical symptoms are often vague and usually relate to abdominal pain and bowel dysmotility (nausea, vomiting, diarrhea, ileus, and constipation) [5]. Complications of PI, including bowel obstruction, volvulus, intussusception, and adhesions following cyst encroachment or collapse, occur in about three percent of patients [6].

Effects of PI outside the bowel wall submucosa typically relate to development of pneumoperitoneum from ruptured cysts in up to nine percent of patients [5]. The development of gas beyond the bowel wall, particularly in otherwise clinically stable patients, is extremely rare. There are no case reports in the literature describing PI in the omentum who were not otherwise moribund from their underlying condition. In this case report, we present a stable patient with extensive PI involving both the colon and omentum two weeks after a small bowel resection for obstruction secondary to adhesions following an abdominoperineal resection two months prior that resolved with judicious conservative management.

## 2. Case Report

A 54-year-old male with no other medical history apart from a 30-pack-year smoking history and a negative contributory family history underwent an open abdominoperineal resection with creation of an end colostomy for a clinical T2 (tumor in the muscularis propria) rectal cancer on magnetic resonance imaging. There were no immediate intraoperative complications and he was discharged home on postoperative day seven. He was then readmitted seven days later with perineal wound dehiscence and a small bowel obstruction in the distal ileum without a clear transition point. The wound dehiscence was treated successfully with debridement and negative pressure wound therapy. The bowel obstruction did not resolve and the patient was placed on total parenteral nutrition. Seven weeks after his initial surgery, he was taken back to the operating room for an exploratory laparotomy and lysis of adhesions. Apart from the distended small bowel, all intra-abdominal organs were healthy and well perfused. This surgery lasted about five hours resulting in several unavoidable small bowel enterotomies in loops of ileum densely adhered in the pelvis. He then required vasopressor therapy and a decision was made to stop the surgery and admit the patient to the intensive care unit overnight for resuscitation. The next afternoon, he was taken back to the operating room where these loops of ileum with enterotomies were resected, and a side-to-side small bowel anastomosis was made between the proximal 250 cm of the small bowel and the distal 20 cm of the terminal ileum. The patient did well and was discharged home ten days later. Final histopathology on the resected small bowel reported acute and chronic inflammation of the mesenteric fat and serosa.

**Figure 1:**
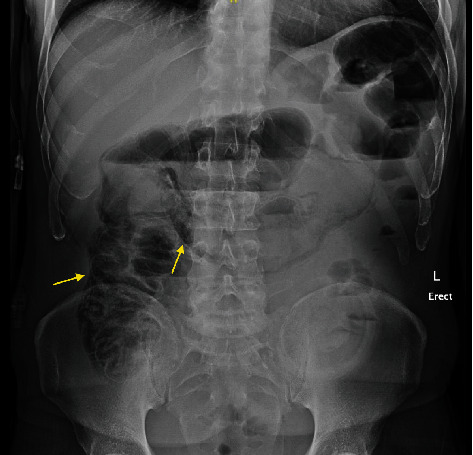
Abdominal X-ray demonstrating extensive pneumatosis intestinalis in the right and transverse colon and greater omentum (yellow arrows). End colostomy stoma bag is seen in the left lower quadrant.

**Figure 2:**
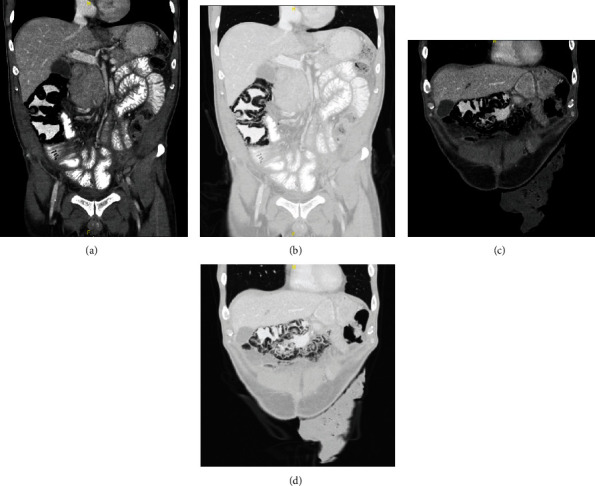
Coronal computer tomography images demonstrating extensive pneumatosis intestinalis in the right and transverse colon and greater omentum. (a) Abdominal window view of the right colon. (b) Lung window view of the right colon. (c) Abdominal window view of the transverse colon and greater omentum. (d) Lung window view of the transverse colon and greater omentum.

**Figure 3:**
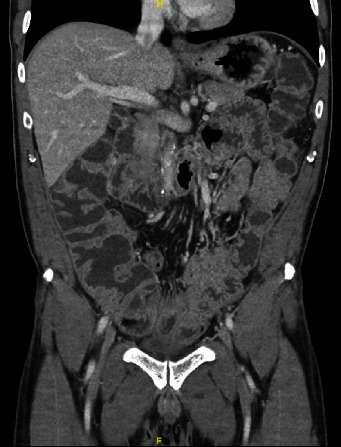
Coronal computer tomography image demonstrating complete resolution of the pneumatosis intestinalis one month following scan in Figure 2.

Two weeks after discharge, he presented for reassessment with a five-day history of intermittent abdominal pain. Physical exam was unremarkable and the stoma was working properly. All vital signs, complete blood cell count, and basic electrolytes panel were within normal limits. Given however his recent history and prolonged hospital stay, we elected to perform imaging to rule out any subclinical causes for this pain. Abdominal X-ray showed extensive PI in the right and transverse colon and greater omentum (Figure 1). Subsequent computer tomography (CT) scan of the abdomen confirmed this finding and did not show another pathology (Figure 2). We elected to admit the patient for observation. He required no intervention given his stable condition. Repeat bloodwork also remained normal. He was discharged home three days later. The patient was again followed up in clinic three days later with a completely benign abdominal exam and resolution of all pain symptoms. Follow-up CT scan one month later showed complete resolution of the PI (Figure 3).

## 3. Discussion

PI is often a complex and multifactorial phenomenon, influenced by a combination of mucosal integrity, intraluminal pressure and gas, and bacterial flora [7]. Its presence in cancer patients is typically seen secondary to processes that induce gastrointestinal mucosal damage such as surgery or chemotherapeutic agents and adjuncts [8]. PI involving the greater omentum is poorly described in the literature. It has been described on autopsy where PI was found throughout the entire intestine and mesentery secondary to gastric carcinoma [9]. Another study reported PI throughout the right and transverse colon with distention of the omentum in the context of septic shock and multiorgan failure [10]. In rats, PI in the omentum and mesentery has been observed following injection of *Clostridium perfringens* into either the small intestine or cecum, or directly in the peritoneal cavity [4]. These experimental findings lend support to the bacterial theory behind PI and could be an explanation for the development of PI in this case report. Our patient had a prolonged operative course with multiple small bowel enterotomies, which would have provided ample opportunity for bacterial seeding. The timing of onset of his PI following surgery is unknown given that it was discovered incidentally on imaging for relatively mild abdominal pain. Operative exploration and intervention was felt to be unduly risky given his stable clinical picture and recent operation two weeks prior. His ultimate stable clinical course supported our use of prudent conservative management. This practice is established for patients with intraluminal PI with otherwise relatively benign symptomatology. Given the multifactorial and often idiopathic nature of PI, the recurrence rate and even the clinical relevance of recurrence are unknown, especially in benign presentations [11]. Hence, follow-up imaging to document resolution is necessary to ensure that potentially treatable causes have been addressed.

## 4. Conclusion

Extraluminal PI outside the portal venous system is virtually unreported in the literature apart from moribund cases. Here, our stable patient presented with imaging findings of PI in the colon and greater omentum, likely secondary to extensive small bowel surgery two weeks prior. The source of the PI is likely multifactorial and may include bacterial seeding and mucosal injury secondary to bowel manipulation and restoration of colonic bowel function after prolonged small bowel obstruction. Clinical stability ultimately directed our conservative management approach. The incidence of extraluminal PI in clinically well postoperative surgical patients is unknown. Ultimately, if PI is discovered incidentally in patients, follow-up with clear instructions for return to medical care is essential until the underlying cause of the PI is treated or imaging demonstrates resolution.

## Data Availability

All data supporting this case report is either presented or cited from previously reported studies and datasets.
